# Supplementary material for: Comparative Transcriptome Analysis Reveals Genetic Mechanisms of Sugarcane Aphid Resistance in Grain Sorghum
Source: Int J Mol Sci. 2021 Jul 1;22(13):7129. doi: 10.3390/ijms22137129 (PMC8268927; doi:10.3390/ijms22137129)
Supplement: Supplementary file 1 [file ijms-22-07129-s001.zip › ijms-1261199-supplementary.pdf]

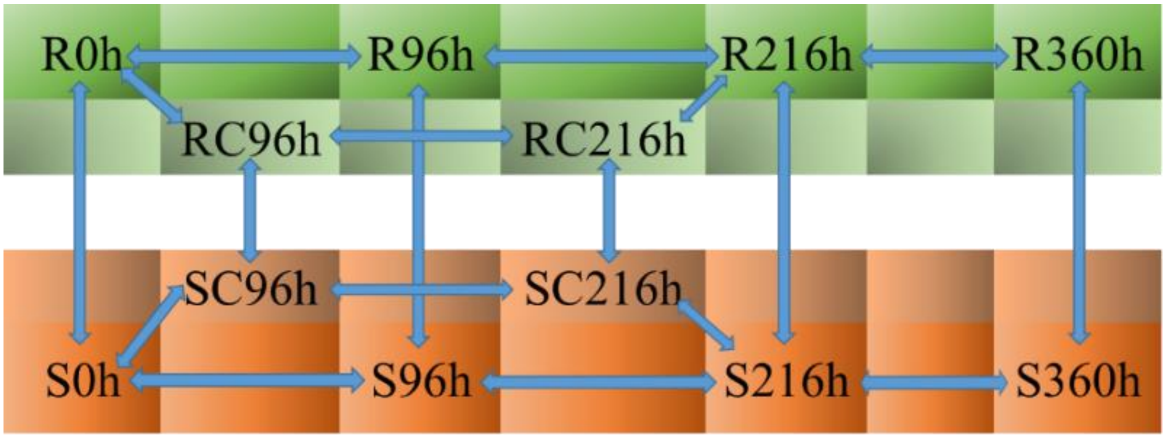

Figure S1: Outline of differential expression analysis of resistant and susceptible sorghum genotypes after SCA infestation

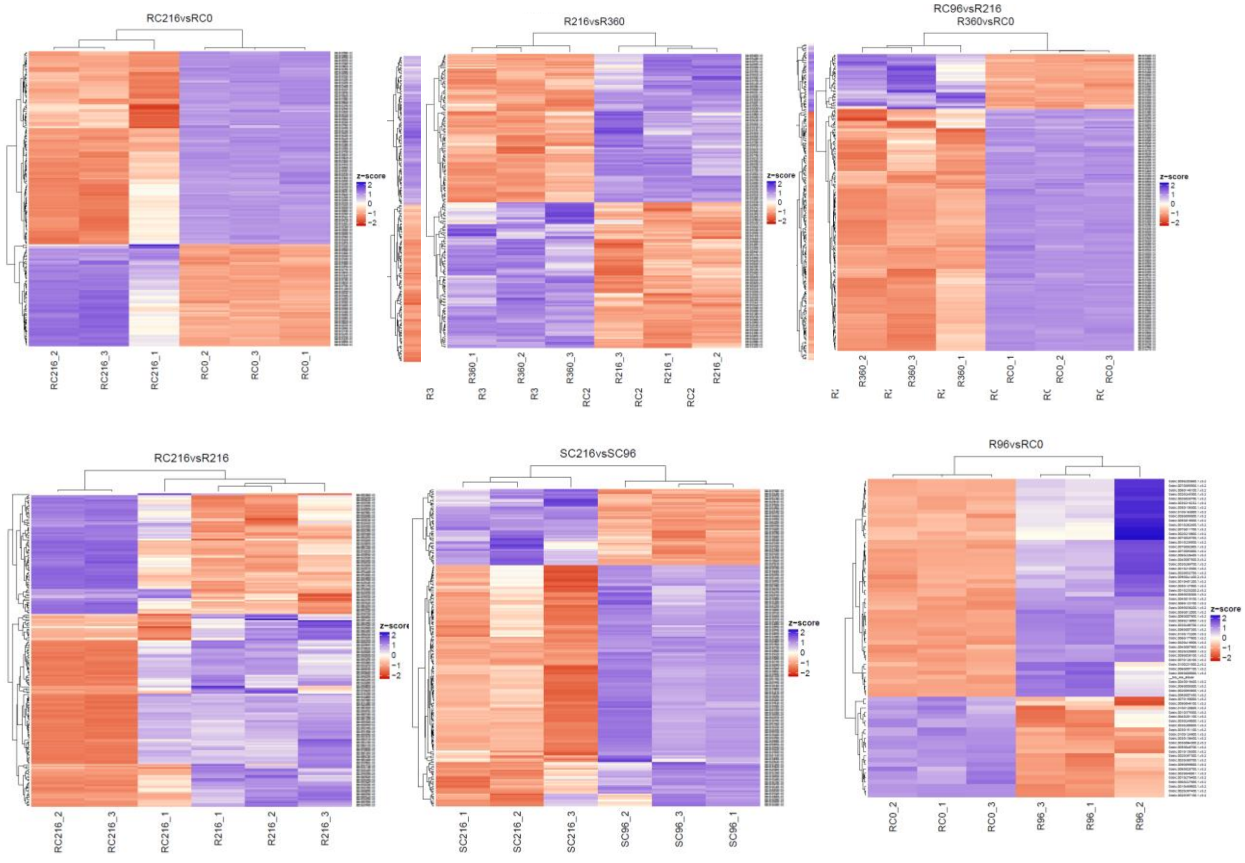

RC216vsRC0
RC216_2
RC216_3
RC216_1
RC0_2
RC0_3
RC0_1
z-score
2
1
0
−1
−2
R216vsR360
R360_1
R360_2
R360_3
R216_3
R216_1
R216_2
RC96vsR216
R360vsRC0
R360_2
R360_3
R360_1
RC0_1
RC0_2
RC0_3
RC216vsR216
RC216_2
RC216_3
RC216_1
R216_1
R216_2
R216_3
SC216vsSC96
SC216_1
SC216_2
SC216_3
SC96_2
SC96_3
SC96_1
R96vsRC0
RC0_2
RC0_1
RC0_3
R96_3
R96_1
R96_2

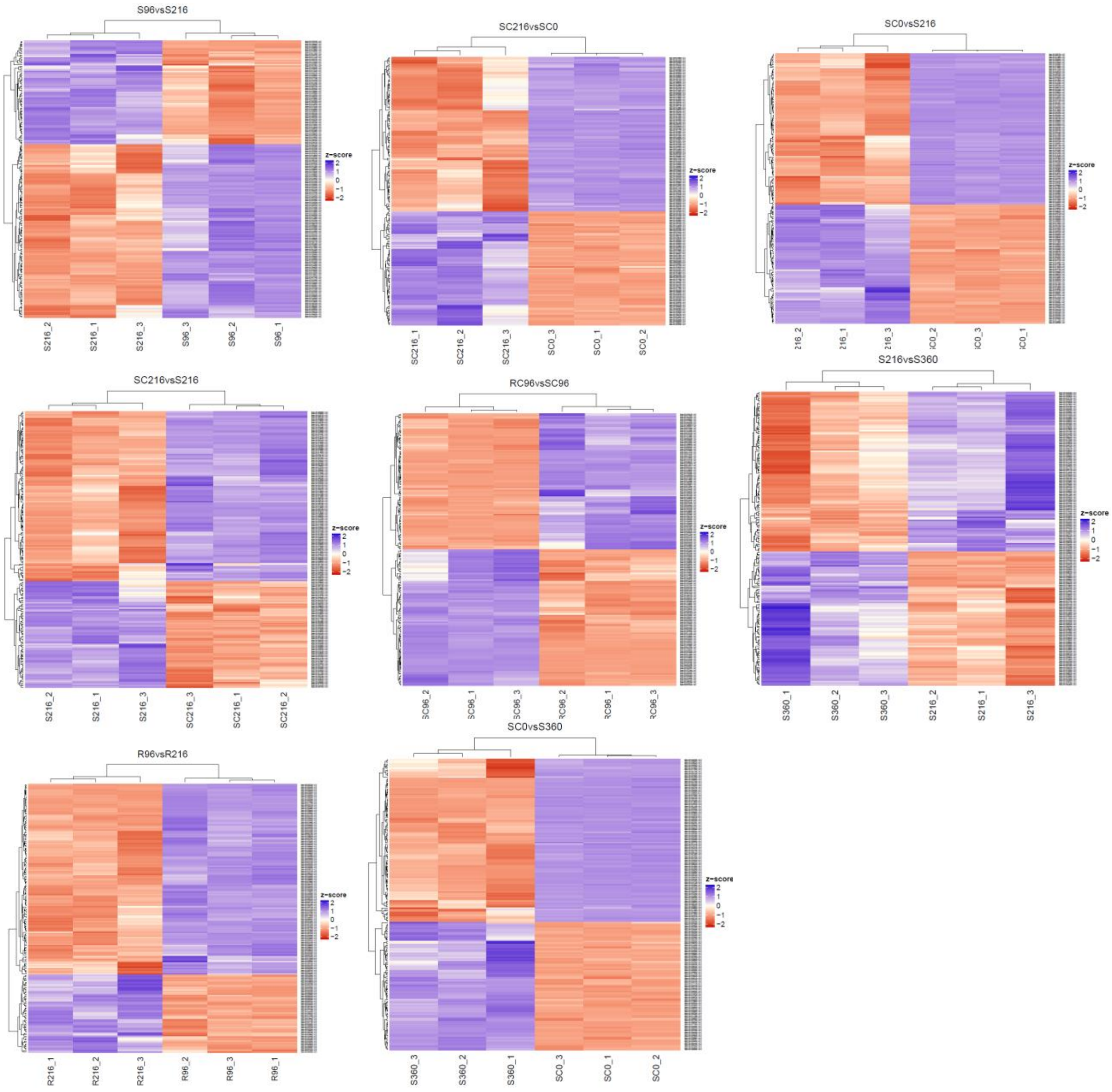

Figure S2: Correlograms plots on the final list of differential expressed genes between pairs of samples

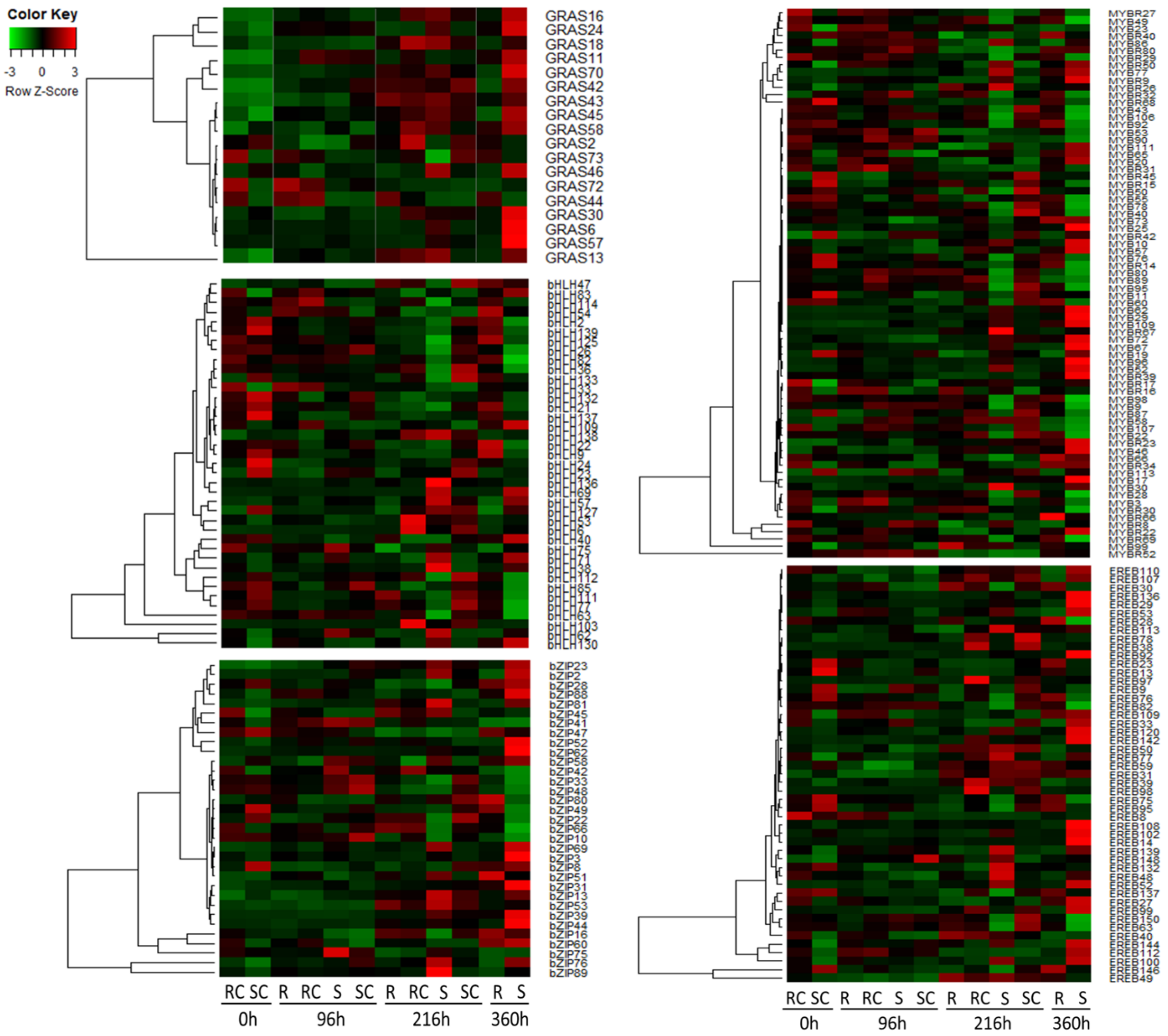

Figure S3: Expression patterns of transcription factors at different time points after SCA infestation in R and S sorghum genotypes

Table S1: Sequence quality of RNA-Seq reads as checked by trimmomatic at minimum length 36 and % mapped to the reference sorghum genome

Samples (3 reps)	Input read pairs	Both surviving	Quality (%)	Forward only	Reverse only	Percent mapping
SC0-1	20869532	20020898	95.93	839518	7578	97.87
SC0-2	21582377	20638744	95.63	935322	6447	97.77
SC0-3	21495627	20578344	95.73	912765	2632	97.62
RC0-1	20804391	19921855	95.76	874370	6220	98.16
RC0-2	23847664	22998304	96.44	834416	13307	98.18
RC0-3	20450650	19625632	95.97	814978	7943	98.32
SC96-1	21612368	20593321	95.28	1011415	5405	97.80
SC96-2	20270518	19402164	95.72	862631	3765	97.69
SC96-3	22932175	21871434	95.37	1053137	5257	97.79
RC96-1	20156506	19352416	96.01	799079	2959	97.38
RC96-2	22339199	21457487	96.05	87660	3148	98.07
RC96-3	21458947	20481303	95.44	970674	4675	97.71
S96-1	20375958	19503653	95.72	854093	16458	96.74
S96-2	20720837	19872620	95.91	870770	5256	97.45
S96-3	20306856	19424981	95.66	870413	9700	95.71
R96-1	21729269	20875591	96.07	847675	3777	96.39
R96-2	22176605	21240941	95.78	924990	8042	97.68
R96-3	20524704	19710937	96.04	805633	5835	96.33
SC216-1	21622958	20666100	95.57	950178	4626	97.84
SC216-2	22273380	21228226	95.31	1037385	5636	97.80
SC216-3	21223391	20263135	95.48	953705	4778	97.64
RC216-1	21405058	20387803	95.25	1011524	3734	98.30
RC216-2	20019075	19182679	95.82	830082	4723	97.61
RC216-3	21648085	20729431	95.76	913309	3464	97.56
S216-1	22475053	21456247	95.47	1011431	5410	93.97
S216-2	21643746	20742298	95.84	894898	4866	96.41
S216-3	20188220	19374798	95.97	807164	4632	93.38
R216-1	22142648	21195608	95.72	938673	5681	97.91
R216-2	20595868	19721048	95.75	860892	12247	97.20
R216-3	21010044	20126127	95.79	873770	7943	96.89
S360-1	22415308	21580073	96.27	821684	11779	86.69
S360-2	21349472	20488840	95.97	852007	6911	92.65
S360-3	21128786	20201576	95.61	920299	4933	95.35
R360-1	22330024	21478556	96.19	840828	8492	97.52
R360-2	21696698	20753367	95.65	936872	4105	97.49
R360-3	20088692	19071273	94.94	1009906	5461	97.73
Average	21358630	20450495	96	878726	6328	95.49

Table S2: Gene ontology annotation of differentially expressed genes in the R genotype in the course of SCA infestation

	GO term	Ontology *	Description	Number in input	Number in Ref	p-value	FDR
R0h	GO:0015979	P	Photosynthesis	34	91	2.1e-23	3.2e-21
R0h	GO:0006091	P	Generation of precursor metabolites and energy	16	98	3e-07	2.2e-05
R0h	GO:0008152	P	Metabolic process	278	7395	6.7e-06	0.00034
R0h	GO:0009579	C	Thylakoid	18	67	5.5e-11	4.7e-09
R0h	GO:0030529	C	Intracellular ribonucleoprotein complex	23	347	0.00085	0.024
R0h	GO:0005840	C	Ribosome	21	295	0.0006	0.024
S0h	GO:0003824	F	Catalytic activity	471	6982	8.8e-08	7.2e-06
S0h	GO:0030246	F	Carbohydrate binding	29	189	4.8e-06	0.00019
S0h	GO:0016740	F	Transferase activity	202	2793	9.8e-05	0.0027
S0h	GO:0000166	F	Nucleotide binding	158	2153	0.00037	0.0075
S0h	GO:0016301	F	Kinase activity	104	1343	0.00083	0.013
S0h	GO:0016772	F	Transferase activity, transferring phosphorus-containing groups	112	1533	0.0033	0.045
R0S0h	GO:0050789	P	Regulation of biological process	46	1432	0.00043	0.021
R0S0h	GO:0019725	P	Cellular homeostasis	10	132	0.00035	0.021
R0S0h	GO:0050794	P	Regulation of cellular process	46	1401	0.00027	0.021
R0S0h	GO:0042592	P	Homeostatic process	10	143	0.00063	0.023
R0S0h	GO:0065007	P	Biological regulation	46	1487	0.00094	0.027
R0S0h	GO:0005576	C	Extracellular region	7	66	0.00043	0.023
RS96h	GO:0007049	P	Cell cycle	8	100	1.8e-06	0.0002
RS96h	GO:0006259	P	DNA metabolic process	10	223	1.3e-05	0.00072
RS96h	GO:0003774	F	Motor activity	11	69	2.4e-11	1.2e-09
RS96h	GO:0003677	F	DNA binding	29	1112	7.6e-09	1.9e-07
RS96h	GO:0017111	F	Nucleoside-triphosphatase activity	16	394	1e-07	1.7e-06
RS96h	GO:0016462	F	Pyrophosphatase activity	16	409	1.7e-07	2.1e-06
RS96h	GO:0016818	F	Hydrolase activity, acting on acid anhydrides, in phosphorus-containing anhydrides	16	418	2.3e-07	2.2e-06
RS96h	GO:0016817	F	Hydrolase activity, acting on acid anhydrides	16	428	3.1e-07	2.5e-06
RS96h	GO:0003676	F	Nucleic acid binding	31	1968	9e-05	0.00064
RS96h	GO:0016787	F	Hydrolase activity	29	2042	0.0009	0.0056
RS96h	GO:0000166	F	Nucleotide binding	28	2153	0.0041	0.023
RS96h	GO:0044446	C	Intracellular organelle part	26	513	4.4e-14	1.3e-12
RS96h	GO:0044422	C	Organelle part	26	515	4.8e-14	1.3e-12
RS96h	GO:0043232	C	Intracellular non-membrane-bounded organelle	26	538	1.3e-13	1.7e-12

RS96h	GO:0043228	C	Non-membrane-bounded organelle	26	538	1.3e-13	1.7e-12
RS96h	GO:0043226	C	Organelle	39	1423	1.7e-12	1.5e-11
RS96h	GO:0043229	C	Intracellular organelle	39	1421	1.6e-12	1.5e-11
RS96h	GO:0005856	C	Cytoskeleton	13	135	1.1e-10	8.8e-10
RS96h	GO:0044424	C	Intracellular part	40	1815	5.8e-10	3.5e-09
RS96h	GO:0005622	C	Intracellular	41	1895	5.6e-10	3.5e-09
RS96h	GO:0005623	C	Cell	42	1999	7.6e-10	3.7e-09
RS96h	GO:0044464	C	Cell part	42	1999	7.6e-10	3.7e-09
RS96h	GO:0032991	C	Macromolecular complex	28	989	2.5e-09	1.1e-08
RS96h	GO:0005634	C	Nucleus	15	669	0.00025	0.001
R96-S96h	GO:0043227	C	Membrane-bounded organelle	15	936	0.0065	0.023
R96-S96h	GO:0043231	C	Intracellular membrane-bounded organelle	15	936	0.0065	0.023
S96h	GO:0006950	P	Response to stress	58	483	6.9e-08	1.6e-05
S96h	GO:0050896	P	Response to stimulus	80	872	6.7e-06	0.00079
S96h	GO:0032501	P	Multicellular organismal process	22	179	0.00063	0.049
S96h	GO:0003824	F	Catalytic activity	443	6982	3.2e-06	0.00026
S96h	GO:0004518	F	Nuclease activity	15	97	0.00057	0.023
S96h	GO:0005576	C	Extracellular region	12	66	0.00056	0.043
R216h	GO:0008152	P	Metabolic process	463	7395	7.2e-05	0.0083
R216h	GO:0009607	P	Response to biotic stimulus	8	20	6.3e-05	0.0083
R216h	GO:0006950	P	Response to stress	47	483	0.00025	0.016
R216h	GO:0051704	P	Multi-organism process	15	88	0.00027	0.016
R216h	GO:0003824	F	Catalytic activity	456	6982	6e-07	4.6e-05
R216h	GO:0030246	F	Carbohydrate binding	24	189	0.00031	0.012
S216h	GO:0003824	F	Catalytic activity	161	6982	1.4e-06	8.7e-05
R360h	GO:0006464	P	Cellular protein modification process	128	1589	2.1e-05	0.0043
R360h	GO:0008152	P	Metabolic process	466	7395	8.5e-05	0.0057
R360h	GO:0043412	P	Macromolecule modification	128	1629	6.1e-05	0.0057
R360h	GO:0009607	P	Response to biotic stimulus	7	20	0.00037	0.019
R360h	GO:0003824	F	Catalytic activity	459	6982	7.1e-07	6.8e-05
R360h	GO:0016301	F	Kinase activity	116	1343	2.9e-06	0.00014
R360h	GO:0016740	F	Transferase activity	205	2793	1.6e-05	0.00049
R360h	GO:0030246	F	Carbohydrate binding	26	189	6.2e-05	0.0015
R360h	GO:0016772	F	Transferase activity, transferring phosphorus-containing groups	121	1533	8.4e-05	0.0016
S360h	GO:0003824	F	catalytic activity	458	6982	0.00019	0.017
S360h	GO:0043232	C	intracellular non-membrane-bounded organelle	52	538	0.00044	0.026
S360h	GO:0043228	C	non-membrane-bounded organelle	52	538	0.00044	0.026

*C=Cellular components, F=Molecular function, and P=biological process
